# Clinical Evaluation of Xpert Xpress CoV-2/Flu/RSV *plus* and Alinity m Resp-4-Plex Assay

**DOI:** 10.3390/diagnostics14070683

**Published:** 2024-03-24

**Authors:** Wai-Sing Chan, Kan-Pui Wong, Siu-Kei Yau, Ching-Yan Wong, Tsz-Ching Chan, Jeffrey Hung, Kristi Tsz-Wan Lai, Chin-Pang Leung, Candy Ling-Na Wang, Chun-Hang Au, Thomas Shek-Kong Wan, Edmond Shiu-Kwan Ma, Bone Siu-Fai Tang

**Affiliations:** Department of Pathology, Hong Kong Sanatorium & Hospital, Hong Kong SAR, China; waising.chan@connect.polyu.hk (W.-S.C.); tommy.ch.au@hksh.com (C.-H.A.); eskma@hksh.com (E.S.-K.M.)

**Keywords:** Xpert Xpress CoV-2/Flu/RSV *plus*, Alinity m Resp-4-Plex, influenza, RSV, SARS-CoV-2

## Abstract

The performance of the Xpert Xpress CoV-2/Flu/RSV *plus* and Alinity m Resp-4-Plex Assays were evaluated using 167 specimens, including 158 human respiratory specimens and 9 external quality assessment program (EQAP) samples. For respiratory specimens, CoV-2/Flu/RSV *plus* exhibited perfect agreement with the standard-of-care (SOC) methods (Cohen’s κ: 1, 100% agreement). The overall positive and negative percent agreement (PPA and NPA) were 100%, with 95% confidence intervals of 96.50 to 100% and 85.70 to 100%, respectively. On the other hand, Resp-4-Plex revealed an almost perfect agreement with the SOC methods (Cohen’s κ: 0.92, 97.71% agreement). The overall PPA and NPA were 100% (95.76 to 100%) and 88.46% (70.20 to 96.82%), respectively. For EQAP samples, the results of CoV-2/Flu/RSV *plus* (9/9) and Resp-4-Plex (4/4) were concordant with the expected results. The experimental limit of detection of severe acute respiratory syndrome coronavirus 2 (SARS-CoV-2) was the lowest (25 copies/mL for both methods), and that of the respiratory syncytial virus was the highest (400 copies/mL for CoV-2/Flu/RSV *plus* and 100 copies/mL for Resp-4-Plex). Threshold cycle (Ct) value correlation showed a large positive linear association between CoV-2/Flu/RSV *plus* and Resp-4-Plex, with R-squared values of 0.92–0.97, and on average, the Ct values of CoV-2/Flu/RSV *plus* were higher than that of Resp-4-Plex by 1.86–2.78, except for Flu A1 target (−0.66). To conclude, the performance of both assay was comparable to the SOC methods for both upper and lower respiratory specimens. Implementation of these rapid assay may reinforce the diagnostic capacity for the post-pandemic co-circulation of SARS-CoV-2 and other respiratory viruses.

## 1. Introduction

The coronavirus disease 2019 (COVID-19) pandemic has lasted more than 3 years and accumulated 774,469,939 reported cases across six World Health Organization regions (as of 10 February 2024) [1]. With the availability of antiviral treatments and the build-up of herd immunity barrier, the global strategy against COVID-19 has been shifting towards ‘living with the virus’ [2,3,4]. Hong Kong has likewise lifted all pandemic control measures since 1 March 2023 [5]. Recently, the resurgence of respiratory viruses other than severe acute respiratory syndrome coronavirus 2 (SARS-CoV-2) has been reported worldwide [6,7,8,9,10,11]. Similarities in clinical presentation make the identification of the culprit and coinfection difficult, posing challenges to diagnosis and patient management. In this regard, rapid, multiplex nucleic acid amplification testing (NAAT) of clinically important respiratory viruses may facilitate timely clinical decision making.

The Xpert Xpress CoV-2/Flu/RSV *plus* and Alinity m Resp-4-Plex Assay are two commercial in vitro diagnostic NAATs under the U.S. Food and Drug Administration’s Emergency Use Authorization. They are designed for the qualitative detection of SARS-CoV-2, influenza A and B viruses, and respiratory syncytial virus (RSV). Xpert Xpress CoV-2/Flu/RSV *plus* targets the nucleocapsid (N), envelope and RNA-dependent RNA polymerase (RdRP) genes of SARS-CoV-2, the matrix (M), basic polymerase, and acidic protein genes of influenza A virus, the M and non-structural (NS) protein genes of influenza B virus and the N genes of RSV A and B [12]. The assay is run on the GeneXpert System which allows for random access of tests with a runtime of about 40 min. The Resp-4-Plex Assay, on the other hand, targets the RdRP and N genes of SARS-CoV-2, the M genes of influenza A virus and RSV, and the NS1 gene of influenza B virus. The assay is run on the Alinity m System, which allows for batch testing of up to 1080 specimens in 24 h, with the first result available in less than 2 h [13].

In this study, we evaluated the performance of the Xpert Xpress CoV-2/Flu/RSV *plus* and Alinity m Resp-4-Plex Assay with a collection of human respiratory specimens, external quality assessment program (EQAP) samples, and an external run control of target viruses.

## 2. Materials and Methods

### 2.1. Specimens

This study was pursued in the Department of Pathology, Hong Kong Sanatorium & Hospital. A total of 167 specimens were tested, including 158 human respiratory specimens collected between October 2022 and February 2024, as well as 9 samples from 2 EQAPs. The respiratory specimens included 121 from the upper and 37 from the lower respiratory tract (URT and LRT). URT specimens comprised 29 nasopharyngeal swabs, 37 combined nasopharyngeal and throat swabs, and 55 combined nasal and throat swabs preserved in a viral transport medium. LRT specimens included 2 endotracheal aspirate, 5 bronchoalveolar lavage fluid, 5 tracheal aspirate, and 25 sputum specimens.

### 2.2. Standard-of-Care (SOC) Methods

The specimens were routinely tested with any of or combination of the SOC methods, including BIOFIRE Respiratory 2.1 *plus* Panel (RP2.1*plus*) or Pneumonia *plus* Panel (PN*plus*) on FilmArray 2.0 or Torch System (BioFire Diagnostics, Salt Lake City, UT, USA); Xpert Xpress SARS-CoV-2 on GeneXpert System (Cepheid, Sunnyvale, CA, USA); and LightMix SarbecoV E-gene plus EAV control (Tib MolBiol, Berlin, Germany) on LightCycler 480 II (Roche, Basel, Switzerland).

### 2.3. Evaluation of Clinical and Analytical Performance

The remnant specimens were tested with both the Xpert Xpress CoV-2/Flu/RSV *plus* (Cepheid, Sunnyvale, CA, USA) and Alinity m Resp-4-Plex Assay (Abbott Molecular, Desplaines, IL, USA) as far as possible, depending on residual quantity. All experimental procedures were performed according to the manufacturers’ recommendations. Cohen’s κ was calculated to measure the overall agreement between CoV-2/Flu/RSV *plus* (or Resp-4-Plex) and the SOC methods [14].

Positive and negative percent agreement (PPA and NPA) of each pathogen target were calculated by using the following formulae:

PPA = TP/(TP + FN) × 100%

NPA = TN/(TN + FP) × 100%

FN, FP, TN, and TP stand for a number of false-negative, false-positive, true-negative, and true-positive specimens, respectively. The 95% confidence intervals (CI) for PPA and NPA were calculated using the online version of GraphPad software (Version 2024) (modified Wald method) [15].

The experimental limit of detection (LoD) was estimated by testing at least quintuple replicates of NATtrol Flu/RSV/SARS-CoV-2 External Run Control & Verification Panel (ZeptoMetrix, Buffalo, NY, USA) at 7 dilutions (10, 25, 50, 100, 200, 300, and 400 copies/mL). This control comprises a mixture of equal concentration (15,000 copies/mL) of influenza A virus (A/Brisbane/10/07), influenza B virus (B/Florida/02/06), RSV B (CH93(18)-18), and SARS-CoV-2 (USA-WA1/2020) [16]. The correlation of threshold cycle (Ct) values between CoV-2/Flu/RSV *plus* and Resp-4-Plex was assessed by considering the R-squared value of respective linear regression.

## 3. Results

### 3.1. Clinical Performance

The results are summarized in Table 1.

For CoV-2/Flu/RSV *plus*, a total of two specimens were excluded from analysis due to inadequacy (specimen 22) and invalid results (specimen 52). For specimen 47, error 5007 was encountered in the first run due to Flu B probe check failure and the repeated run was valid. There were 2 invalid runs (0.83%) out of a total of 240.

CoV-2/Flu/RSV *plus* exhibited perfect agreement with the SOC methods (Cohen’s κ: 1, 100% agreement). The overall PPA and NPA were 100%, with a 95% CI of 96.50 to 100% and 85.70 to 100%, respectively.

For Resp-4-Plex, a total of 27 specimens were excluded from analysis due to inadequacy (25 specimens) and invalid results (2 specimens). The error rate was 1.07% (2 out of 187 runs). 

Resp-4-Plex displayed almost perfect agreement with the SOC methods, with Cohen’s κ of 0.92 and 97.71% overall agreement. The overall PPA and NPA were 100% (95.76 to 100%) and 88.46% (70.20 to 96.82%), respectively.

Specimens 30, 32, 33, and 34 were positive for SARS-CoV-2 variant XBB.1 with C26270T and A26275G mutations detected in the E gene. The E gene Ct values of the SOC method Xpert Xpress SARS-CoV-2 were 0, and N2 Ct values ranged from 25.8 to 34.3. SARS-CoV-2 was detected by both CoV-2/Flu/RSV *plus* and Resp-4-Plex in these four specimens.

For the three specimens positive for two viruses (specimen 106, SARS-CoV-2 and influenza A virus; specimen 107, SARS-CoV-2 and RSV; specimen 108, influenza A virus and RSV), the results of CoV-2/Flu/RSV *plus* and Resp-4-Plex were concordant with that of the SOC methods.

For Resp-4-Plex, the results of three specimens were discordant with CoV-2/Flu/RSV *plus* and the SOC method RP2.1*plus*. These included specimens 117 (RSV detected by Resp-4-Plex), 122, and 124 (SARS-CoV-2 detected by Resp-4-Plex). The Ct values ranged from 35.96 to 38.34. CoV-2/Flu/RSV *plus* and RP2.1*plus* were negative for all four virus targets.

### 3.2. EQAP Samples

Table 2 summarizes the results of EQAP samples. The samples were obtained from two EQAPs. Nine samples were tested by CoV-2/Flu/RSV *plus*. Only four samples were tested by Resp-4-Plex, as the other five samples were not sufficient for testing. The results of both assay were concordant with the expected answers. The Ct values of Resp-4-Plex appeared to be lower than those of CoV-2/Flu/RSV *plus*.

### 3.3. Experimental LoD and Precision

Table 3 summarizes the detection rate at each dilution of the reference material and precision in terms of the coefficient of variation (CV) in Ct values. The LoD for simultaneous detection of all four viruses was 400 copies/mL for CoV-2/Flu/RSV *plus* and 100 copies/mL for Resp-4-Plex. For individual viruses, the LoD of SARS-CoV-2, influenza A and B viruses, and RSV were 25, 100, 200, and 400 copies/mL, respectively, for CoV-2/Flu/RSV *plus*, and 25, 50, 50, and 100 copies/mL, respectively, for Resp-4-Plex. The average CV at or above the LoD was 1.61% (0.70–2.63%) for CoV-2/Flu/RSV *plus* and 1.56% (0.57–3.76%) for Resp-4-Plex.

### 3.4. Inter-Assay Correlation of Ct Values

**Figure 1:**
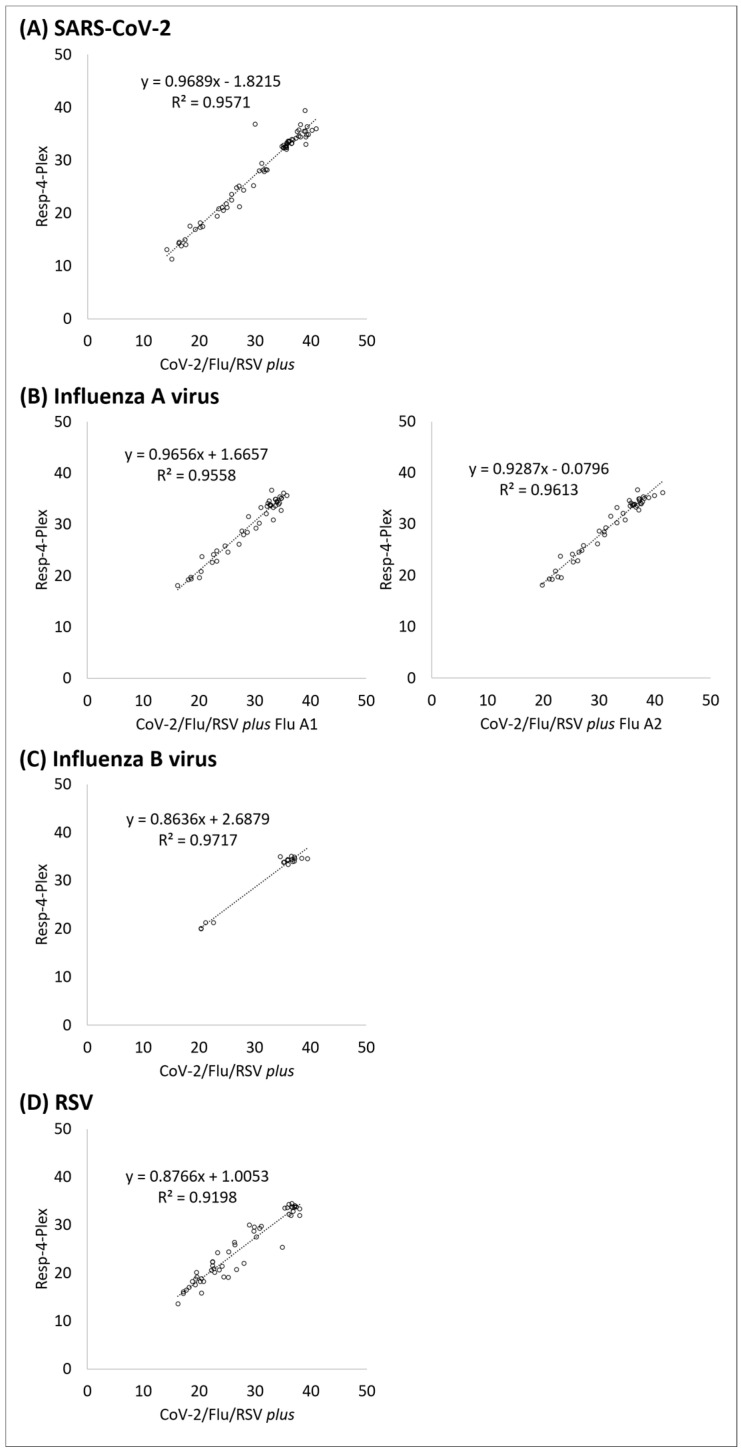
Correlation of Ct values between Xpert Xpress CoV-2/Flu/RSV *plus* (x axis) and Resp-4-Plex Assay (y axis) for (**A**) SARS-CoV-2, (**B**) influenza A virus, (**C**) influenza B virus, and (**D**) RSV.

Figure 1 shows the correlation of Ct values between CoV-2/Flu/RSV *plus* and Resp-4-Plex. The data points were generated from clinical specimens, EQAP samples, and dilutions of reference material at or above the experimental LoD of individual viruses. R-squared values for SARS-CoV-2, the two targets of influenza A virus (Flu A1 or Flu A2 of CoV-2/Flu/RSV *plus* versus Flu A of Resp-4-Plex), influenza B virus, and RSV, were 0.96, 0.96, 0.96, 0.97, and 0.92 with average Ct value differences of 2.78 ± 1.60 (−6.87 to 6.03), −0.66 ± 1.20 (−3.77 to 2.45), 2.38 ± 1.19 (−0.66 to 5.21), 1.86 ± 1.28 (−0.36 to 4.93), and 2.37 ± 2.05 (−0.99 to 9.62), respectively.

## 4. Discussion

Our data showed that the clinical performance of CoV-2/Flu/RSV *plus* and Resp-4-Plex were comparable to the SOC assay. Commercial PCR panels like RP2.1*plus* and PN*plus* are the choices for broad-range detection; CoV-2/Flu/RSV *plus* and Resp-4-Plex, on the other hand, focus on the four clinically important respiratory viruses. In our laboratory, the positive rate of CoV-2/Flu/RSV *plus* during the winter surge of influenza and COVID-19 was 38.38% (January and February 2024). The time lapse between specimen receipt by laboratory staff and result reporting was 1.67 h on average. The data suggest that the rapid PCR testing of the four viruses may deliver actionable results to physicians within a reasonable timeframe for a significant portion of symptomatic patients, and this may, in turn, lessen the test burden on broad-range PCR instruments. Notably, Resp-4-Plex is less favorable than CoV-2/Flu/RSV *plus* in turnaround time, yet its high sample throughput is a merit for laboratories with massive test volumes.

Interestingly, from our CoV-2/Flu/RSV *plus* data, the SARS-CoV-2-influenza virus combination comprised the majority (80.00%) of codetections in the post-COVID-19 era. From the literature, the coinfection of SARS-CoV-2 and influenza viruses significantly increased the odds of receiving invasive mechanical ventilation and in-hospital mortality in hospitalized populations [17]. Another study by Pawlowski and coworkers revealed that ‘flurona’ was rare and mostly observed in relatively young and healthy patients without an increased risk of hospitalization, intensive care unit (ICU) admission, or death, yet with more emblematic viral symptoms [18]. The authors remarked that the clinical outcome of coinfection in patients with risk factors for severe COVID-19 required further observation. A systematic review and meta-analysis of 95 studies revealed that the odds ratios for ICU admission, mechanical ventilation support, and mortality were significantly higher among COVID-19 patients coinfected with influenza A virus than with mono-infections [19]. These findings underscore the importance of rapid PCR testing as well as seasonal COVID-19 and influenza vaccination for high-risk individuals.

There were three discordant results for Resp-4-Plex. The specimens revealed late amplification, and the comparator assay were negative for the targets detected. We had insufficient clinical and epidemiological data to interpret the validity of these positive signals. This reflects the need for a retest algorithm and clinician advice for weak positive results in real-life practice.

An interesting observation was identified about the positive cutoff values of CoV-2/Flu/RSV *plus*. Based on the LoD data, it appeared that the positive cutoff value of RSV was much higher than other targets. For instance, we encountered a ‘negative’ RSV result with a Ct value of 38.1, fluorescence intensity (EndPt) of 257 with a sigmoidal amplification curve, and ‘positive’ results of other targets with Ct values of 39.3 to 44.1 and EndPt of 46 to 150. In this regard, checking the amplification curves can help avoid false negative results.

The performance of CoV-2/Flu/RSV *plus* and Resp-4-Plex have also been evaluated in other studies. For instance, Noble and coworkers compared the performance of CoV-2/Flu/RSV *plus* with various SOC assay [20]. A total of 97 respiratory specimens were tested. The error rate was 0.85% (1/118) which was similar to this study (0.83%). The overall PPA and NPA were 100% and 83.3%, respectively. The authors commented that the NPA was negatively influenced by the low number of negative specimens (*n* = 12). The authors also reported that CoV-2/Flu/RSV *plus* was compatible with SARS-CoV-2 variants, including Omicron BA.1. Our data also showed that both CoV-2/Flu/RSV *plus* and Resp-4-Plex were compatible with SARS-CoV-2 XBB.1 variant. In another study by Johnson and coworkers, CoV-2/Flu/RSV *plus* was compared with its prior version, Xpert Xpress CoV-2/Flu/RSV, with 171 clinical specimens, including 11 mixed specimens, to assess target interaction [21]. The authors reported that the percentage of agreement for SARS-CoV-2, influenza A and B viruses, and RSV were 100%, 100%, 99.4%, and 100%, respectively. CoV-2/Flu/RSV *plus* was also inclusive for SARS-CoV-2 variants Omicron BA.1 and BA.2. Zhen and colleagues evaluated the analytical performance of Resp-4-Plex and compared its performance to various assay [22]. Resp-4-Plex exhibited 100% PPA for influenza A virus and RSV and 95% for SARS-CoV-2 and influenza B virus. The NPA for all four viruses was 100%. The LoD was 26 ± 21 copies/mL for SARS-CoV-2, 36 ± 22 copies/mL for influenza A virus, 22 ± 22 copies/mL for influenza B virus, and 22 ± 23 copies/mL for RSV, which were lower than the LoDs estimated in this study except for SARS-CoV-2. Quinton and colleagues compared the performance of Resp-4-Plex and NeuMoDx Flu A-B/RSV/SARS-CoV-2 Vantage in a study involving 181 remnant specimens [23]. They reported that both assay showed 100% total agreement with the SOC assay. The overall precision of both assay was 100%. All in all, the performance of CoV-2/Flu/RSV *plus* and Resp-4-Plex was verified in a number of studies. 

Our study had two major constraints. First, not all remnant specimens were sufficient for parallel testing by both methods. In particular, the number of influenza B virus-positive specimens for Resp-4-Plex was suboptimal (n = 4). Second, more LRT specimens should be tested to thoroughly assess the performance of both assay.

## 5. Conclusions

We evaluated two representative types of ‘random-access’ molecular diagnostic assay for multiplex detection of four clinically important respiratory viruses, each of which is merited by short assay time or high sample throughput. These merits may allow for more flexibility to cope with the sharp rise in test burden and stat specimens during the co-circulation of SARS-CoV-2 and other respiratory viruses.

## Figures and Tables

**Table 1:** Performance of Xpert Xpress CoV-2/Flu/RSV *plus* and Alinity m Resp-4-Plex on clinical specimens.

Pathogen Targets	FN	FP	TN	TP	PPA	95% CI for PPA (%)	NPA	95% CI for NPA (%)	Remarks
**Xpert Xpress CoV-2/Flu/RSV *plus***									For CoV-2/Flu/RSV *plus*, 1 specimen was insufficient for testing and the result of 1 specimen was invalid. Both specimens were excluded from analysis. For Resp-4-Plex, 25 specimens were insufficient for testing, and the results of 2 specimens were invalid. A total of 27 specimens were excluded from analysis. Specimens 30, 32, 33, and 34 were positive for SARS-CoV-2 variant XBB.1, with C26270T and A26275G mutations detected in the E gene. The E gene Ct values of the SOC Xpert Xpress SARS-CoV-2 were 0. The N2 Ct values ranged from 25.8 to 34.3. For CoV-2/Flu/RSV *plus*, error 5007 was encountered in the first run of specimen 47, and the repeated run was valid. SARS-CoV-2 and influenza A virus were codetected in specimen 106. SARS-CoV-2 and RSV were codetected in specimen 107. Influenza A virus and RSV were codetected in Specimen 108. For Resp-4-Plex, specimen 117 was positive for RSV (Ct value: 35.96). Specimens 122 and 124 were positive for SARS-CoV-2 (Ct values: 38.34 and 37.74). CoV-2/Flu/RSV *plus* and the SOC method RP2.1*plus* were negative for all 4 virus targets. **CoV-2/Flu/RSV *plus*:** **Percentage of agreement: 100%** **Cohen’s κ: 1 (perfect agreement)** **Resp-4-Plex:** **Percentage of agreement: 97.71%** **Cohen’s κ: 0.92 (almost perfect agreement)**
SARS-CoV-2	0	0	117	39	100	89.32 to 100.00	100	96.18 to 100.00	
Influenza A virus	0	0	130	26	100	84.76 to 100.00	100	96.55 to 100.00	
Influenza B virus	0	0	133	23	100	83.09 to 100.00	100	96.63 to 100.00	
RSV	0	0	116	40	100	89.56 to 100.00	100	96.15 to 100.00	
**Overall**	**0**	**0**	**28**	**128**	**100**	**96.50 to 100.00**	**100**	**85.70 to 100.00**	
**Alinity m Resp-4-Plex**									
SARS-CoV-2	0	2	91	38	100	89.07 to 100.00	97.85	92.03 to 99.88	
Influenza A virus	0	0	105	26	100	84.76 to 100.00	100	95.76 to 100.00	
Influenza B virus	0	0	127	4	100	45.41 to 100.00	100	96.47 to 100.00	
RSV	0	1	93	37	100	88.80 to 100.00	98.94	93.64 to >99.99	
**Overall**	**0**	**3**	**23**	**105**	**100**	**95.76 to 100.00**	**88.46**	**70.20 to** **96.82**	

CI, confidence interval; Ct, threshold cycle; FN, false negative; FP, false positive; NPA, negative percent agreement; PPA, positive percent agreement; RSV, respiratory syncytial virus; SARS-CoV-2, severe acute respiratory syndrome coronavirus 2; SOC, standard-of-care; TN, true negative; TP, true positive.

**Table 2:** Performance of Xpert Xpress CoV-2/Flu/RSV *plus* and Alinity m Resp-4-Plex on EQAP samples.

Samples	Expected Results	Xpert Xpress CoV-2/Flu/RSV *plus* Ct Values					Resp-4-Plex Ct Values			
		SARS-CoV-2	Flu A1	Flu A2	Flu B	RSV	COV2	FLUA	FLUB	RSV
EQAP 1, sample 1	SARS-CoV-2	26	0	0	0	0	25.09	ND	ND	ND
EQAP 1, sample 2	Not detected	0	0	0	0	0	ND	ND	ND	ND
EQAP 1, sample 3	SARS-CoV-2	28.8	0	0	0	0	28.1	ND	ND	ND
EQAP 1, sample 4	SARS-CoV-2	33.1	0	0	0	0	32.05	ND	ND	ND
EQAP 2, sample 1	Influenza A virus	0	19.1	21.6	0	0	The samples were not sufficient for testing by Resp-4-Plex			
EQAP 2, sample 2	RSV	0	0	0	0	22.7				
EQAP 2, sample 3	Not detected	0	0	0	0	0				
EQAP 2, sample 4	SARS-CoV-2	28.6	0	0	0	0				
EQAP 2, sample 5	Influenza B virus	0	0	0	18.6	0				

ND, not detected.

**Table 3:** Detection rate and precision for each dilution of the reference material.

Virus Concentration (Copies/mL)	Target	Xpert Xpress CoV-2/Flu/RSV *plus*			Resp-4-Plex		
		# Proportion Detected	Ct Value Range	* CV	# Proportion Detected	Ct Value Range	* CV
10	SARS-CoV-2	4/5	41.2–44.1	N/A	3/5	36.18–36.85	N/A
	Flu A	1/5	FluA1: 38.2–44.5 FluA2: N/A	N/A	3/5	36.99–39.44	N/A
	Flu B	0/5	N/A	N/A	2/5	37.55–38	N/A
	RSV	0/5	38.7 for 1 replicate	N/A	3/5	37.68–37.98	N/A
25	SARS-CoV-2	5/5	37.8–40.2	2.21%	5/5	35.77–39.52	3.76%
	Flu A	4/5	FluA1: 34.9–38.7 FluA2: 37.2–41.8	N/A	3/5	36.76–38.14	N/A
	Flu B	2/5	37.5–43.4	N/A	1/5	37.97 for 1 replicate	N/A
	RSV	2/5	36.7–41.5	N/A	1/5	36.05 for 1 replicate	N/A
50	SARS-CoV-2	5/5	37.8–39.5	1.72%	5/5	34.46–34.94	0.58%
	Flu A	4/5	FluA1: 34.1–38.8 FluA2: 37.4–42.1	N/A	5/5	35.55–37.12	N/A
	Flu B	2/5	38.6–42.9	N/A	5/5	35.47–37.74	N/A
	RSV	0/5	39.9–43.7	N/A	4/5	35.55–36.2	N/A
100	SARS-CoV-2	5/5	36.1–37.3	1.07%	20/20	33.24–35.88	1.71%
	Flu A	5/5	FluA1: 33–35.8 FluA2: 36.9–39.9	2.60% 2.63%	20/20	33.98–38.01	2.91%
	Flu B	4/5	35.9–37.7	N/A	20/20	34.42–36.79	1.82%
	RSV	2/5	37.7–43.2	N/A	20/20	33.71–37.96	3.17%
200	SARS-CoV-2	20/20	35.2–36.7	1.04%	5/5	32.95–33.72	0.87%
	Flu A	20/20	FluA1: 32.8–34.4 FluA2: 36.5–38.8	1.28% 1.46%	5/5	34.04–35.01	0.97%
	Flu B	20/20	35.9–39.5	2.21%	5/5	34.37–35.1	0.75%
	RSV	15/20	36.3–39.6	N/A	5/5	33.71–34.41	0.78%
300	SARS-CoV-2	13/13	35–35.7	0.70%	5/5	32.18–33.36	1.21%
	Flu A	13/13	FluA1: 32.2–34.2 FluA2: 35.6–37.6	1.55% 1.72%	5/5	33.4–34.98	1.66%
	Flu B	13/13	35.1–37.2	1.54%	5/5	33.97–34.56	0.57%
	RSV	11/13	36.1–38.2	N/A	5/5	32.23–34.29	2.03%
400	SARS-CoV-2	20/20	34.4–35.7	0.85%	5/5	32.37–32.95	0.64%
	Flu A	20/20	FluA1: 32.3–33.3 FluA2: 35.4–37.8	0.87% 1.68%	5/5	33.87–34.7	0.89%
	Flu B	20/20	34.6–37.1	1.59%	5/5	33.32–34.96	1.59%
	RSV	20/20	35.2–38	2.30%	5/5	31.98–33.61	2.18%

* CV, coefficient of variation. #, Proportion detected is represented by ‘number of detected replicates/total number of replicates tested’. N/A, not applicable.

## Data Availability

The data presented in this study are available upon request from the corresponding author.
